# MicroRNA-135a Protects Against Ethanol-Induced Apoptosis in Neural Crest Cells and Craniofacial Defects in Zebrafish by Modulating the Siah1/p38/p53 Pathway

**DOI:** 10.3389/fcell.2020.583959

**Published:** 2020-10-02

**Authors:** Fuqiang Yuan, Yang Yun, Huadong Fan, Yihong Li, Lanhai Lu, Jie Liu, Wenke Feng, Shao-yu Chen

**Affiliations:** ^1^Department of Pharmacology and Toxicology, University of Louisville Health Sciences Center, Louisville, KY, United States; ^2^University of Louisville Alcohol Research Center, Louisville, KY, United States; ^3^College of Environment and Resource, Research Center of Environment and Health, Shanxi University, Taiyuan, China; ^4^Department of Medicine, University of Louisville, Louisville, KY, United States

**Keywords:** miRNA-135a, ethanol, apoptosis, neural crest cells, Siah1, craniofacial defects, zebrafish

## Abstract

MicroRNAs (miRNAs) are small non-coding RNAs that are involved in various biological processes, including apoptosis, by regulating gene expression. This study was designed to test the hypothesis that ethanol-induced downregulation of miR-135a contributes to ethanol-induced apoptosis in neural crest cells (NCCs) by upregulating Siah1 and activating the p38 mitogen-activated protein kinase (MAPK)/p53 pathway. We found that treatment with ethanol resulted in a significant decrease in miR-135a expression in both NCCs and zebrafish embryos. Ethanol-induced downregulation of miR-135a resulted in the upregulation of Siah1 and the activation of the p38 MAPK/p53 pathway and increased apoptosis in NCCs and zebrafish embryos. Ethanol exposure also resulted in growth retardation and developmental defects that are characteristic of fetal alcohol spectrum disorders (FASD) in zebrafish. Overexpression of miRNA-135a significantly reduced ethanol-induced upregulation of Siah1 and the activation of the p38 MAPK/p53 pathway and decreased ethanol-induced apoptosis in NCCs and zebrafish embryos. In addition, ethanol-induced growth retardation and craniofacial defects in zebrafish larvae were dramatically diminished by the microinjection of miRNA-135a mimics. These results demonstrated that ethanol-induced downregulation of miR-135a contributes to ethanol-induced apoptosis in NCCs by upregulating Siah1 and activating the p38 MAPK/p53 pathway and that the overexpression of miRNA-135a can protect against ethanol-induced apoptosis in NCCs and craniofacial defects in a zebrafish model of FASD.

## Introduction

Fetal alcohol spectrum disorder (FASD) is an umbrella term used to describe the range of disorders that occur in an individual whose mother drinks alcohol during pregnancy. Individuals with FASD may have abnormal facial features, growth retardation, central nervous system dysfunction, and learning disabilities (Mukherjee et al., 2006; Mantha et al., 2014). Studies have shown that the ethanol-induced apoptosis in neural crest cells (NCCs), a multipotent progenitor cell population that can give rise to a diversity of cell types, including mesenchymal cells that form craniofacial cartilages, bones, and dermis (Smith, 1997; Dash and Trainor, 2020), is one of the major components of the pathogenesis of FASD (Cartwright and Smith, 1995a; Muralidharan et al., 2013). Multiple signaling pathways have been reported to be involved in ethanol-induced apoptosis, including B-cell lymphoma 2 (Bcl-2) (Hong et al., 2002), p53 (Jana et al., 2010), nuclear factor (erythroid-derived 2)-like 2 (Nrf2) (Chen et al., 2013a), p38 mitogen-activated protein kinase (MAPK), and seven *in absentia* homolog 1 (Siah1) (Yuan et al., 2017).

Seven *in absentia* homolog 1 is a member of a highly conserved family of E3 ubiquitin ligases (Carthew and Rubin, 1990) that is widely expressed in mouse embryos and adult tissues (Della et al., 1993). Siah plays a critical role in a variety of biological processes, including apoptosis (Nemani et al., 1996). Our previous study has demonstrated that ethanol treatment can significantly increase the expression and nuclear translocation of Siah1 in NCCs and that Siah1 signaling plays a critical role in ethanol-induced apoptosis in NCCs (Sun et al., 2014). We have also shown that ethanol-induced upregulation of Siah1 can induce apoptosis in NCCs through p38 MAPK-mediated activation of the p53 signaling pathway (Yuan et al., 2017). However, the mechanisms by which ethanol upregulates Siah1 in NCCs are not clear.

MicroRNAs are small non-coding RNAs that are involved in various physiological and pathological processes, including apoptosis, by regulating gene expression. MiR-135a, a member of the miR-135 superfamily, has been reported to be involved in the tumorigenesis and act as a tumor suppressor (Wu et al., 2012; Kroiss et al., 2015; Xu et al., 2016). MiR-135a expression in astrocytes has also been linked to brain inflammation and angiogenesis in Alzheimer’s disease (Ko et al., 2015). Studies have also shown that miR-135a can promote proliferation and inhibit apoptosis of astrocytes in the bacterial meningitis rat models (Dong et al., 2019). Overexpression of miR-135a can also protect human umbilical vein endothelial cells against mechanical stretch-induced increases in apoptosis and ventilator-induced lung injury (Yan et al., 2018). In addition, Siah1 has been identified as a target of miR-135a in HeLa cells and mouse zygotes (Pang et al., 2011). It has been reported that miR-135a can upregulate β-catenin in cervical epithelial cells by targeting Siah1 (Leung et al., 2014).

Zebrafish is a well-established animal model for biomedical research, including FASD research (Bilotta et al., 2004) and an excellent animal model to study gene-ethanol interactions (McCarthy et al., 2013). The rapid external development of zebrafish embryos coupled with their transparency allows rapid analysis of structure and function in the intact embryos (Beis and Stainier, 2006). Studies have demonstrated that embryonic exposure to ethanol resulted in dysmorphology and behavioral deficits that parallel those of FASD in zebrafish (Bilotta et al., 2004; Carvan et al., 2004; Fernandes and Gerlai, 2009; Wang et al., 2018) and that the developmental defects induced by embryonic ethanol exposure can be examined in zebrafish larvae by measuring the eye diameter (Bilotta et al., 2004), body length, and craniofacial cartilage (Marrs et al., 2010).

In the present study, an *in vitro* model of NCCs and a zebrafish model of FASD were used to elucidate the mechanisms by which miR-135a modulates ethanol-induced apoptosis in NCCs and craniofacial defects in zebrafish embryos. We found that ethanol treatment decreased the expression of miR-135a and thereby increased the expression of its direct target, Siah1, which, in turn, activated the p38 MAPK/p53 pathway, increased apoptosis in NCCs and zebrafish embryos and resulted in growth retardation and developmental defects. Overexpression of miR-135a significantly reduced ethanol-induced upregulation of Siah1 and the activation of the p38 MAPK/p53 pathway, decreased ethanol-induced apoptosis in NCCs and zebrafish embryos, and diminished ethanol-induced growth retardation and dysmorphology in zebrafish larvae. These results demonstrate that ethanol-induced downregulation of miR-135a contributes to ethanol-induced apoptosis in NCCs and developmental defects in zebrafish embryos by upregulating Siah1 and activating the p38 MAPK/p53 pathway.

## Materials and Methods

### Cell Culture and Ethanol Treatment

Neural crest cells (JoMa1.3 cells) were cultured as described previously (Maurer et al., 2007; Chen et al., 2013a). Cells were grown on cell culture plates/dishes coated with fibronectin and maintained in Dulbecco’s modified Eagle’s medium (DMEM): Ham’s F12 (1:1) at 37°C in 5% CO_2_. NCCs were exposed to medium containing 50 mM ethanol for 24 h. The stable ethanol levels were maintained by using the methods described previously (Yan et al., 2010).

### Zebrafish Maintenance and Ethanol Treatment

Adult AB zebrafish (*Danio rerio*) were obtained from the Zebrafish International Resource Center (ZIRC) at the University of Oregon, Eugene, OR, United States and maintained in 14 h:10 h light: dark cycles at 28°C. Fertilized eggs were collected after natural spawning and used for this study. For ethanol treatment, the zebrafish embryos at 3 hours post-fertilization (hpf) were treated with 0, 1, or 1.5% ethanol. At 24 hpf, the embryos were either collected for molecular analysis or transferred to fresh system water. These embryos transferred to fresh system water were collected at 4 or 5 days post-fertilization (dpf) for morphological analysis. This study was approved by the Institutional Animal Care and Use Committee of the University of Louisville.

### Microinjection of miRNA Mimics Into Zebrafish Embryos

Zebrafish embryos were microinjected at 1 hpf with 2 nl of synthetic miRNA-135a mimics and control mimics (10 μM) prepared with 1 × Danieau solution [58 mM NaCl, 0.7 mM KCl, 0.4 mM MgSO_4_, 0.6 mM Ca(NO_3_)_2_, 5.0 mM HEPES, pH 7.6] by using a PLI-100A Plus Picoinjector (Harvard Apparatus, Holliston, MA, United States), as previously described (Pineda et al., 2005; Bill et al., 2009; Su et al., 2014). Successful injections were monitored via the co-injection with 0.1% Fast Green. After microinjection, embryos were transferred to a petri dish containing ethanol solution or system water for treatment from 3 to 24 hpf, as described above. Zebrafish embryos were collected at 24 hpf for analysis of the expression of miR-135a and Siah1, and the analysis of apoptosis. Zebrafish larvae were collected at 4 and 5 dpf for morphological analysis and craniofacial cartilage defect analysis, respectively.

### Analysis of miRNA Expression

The expression of miRNA-135a in NCCs and zebrafish embryos was determined as previously described (Chen et al., 2015). Total RNA was isolated with mirVana miRNA Isolation Kit (Ambion, Austin, TX, United States), and quantitative RT-PCR was performed by using TaqMan MicroRNA assays (Ambion, Austin, TX, United States), following the manufacturer’s instructions. All TaqMan microRNA assays were performed in triplicate. Data were normalized with snoRNA202 (NCCs) or U6 snRNA (zebrafish embryos) as endogenous controls. The relative expression of miR-135a was calculated using the comparative threshold cycle (Ct) method as described previously (Chen et al., 2015).

### miRNA Mimics and Inhibitors Transfection

For transient transfection, miRNA-135a mimics, miRNA inhibitors, control mimics, or control inhibitors at a final concentration of 50 nM were transfected into NCCs using Lipofectamine 2000 (Life Technologies, Grand Island, NY, United States), following the manufacturer’s instructions. The cells were harvested 48 h after transfection for additional treatments and analysis.

### Construction of Luciferase Reporter Plasmids and Reporter Assays

MiRNA-135a target sites in the 3′-untranslated regions (3′-UTRs) of mouse Siah1 mRNA were predicted by using Target Scan^1^ and microRNA^2^, and compared with the target sites reported in the previous study (Leung et al., 2014). The 3′-UTR of Siah1 containing putative miR-135a binding sites was amplified from mouse genomic DNA and cloned into the pMIR-REPORT^TM^ (Ambion, Austin, TX, United States). Primers used to clone the DNA fragments containing the Siah1 3′-UTR were: 5′ gactACTAGTtttcttttaactgacaagccatctgcgtggtcatagAAGCTTgcta 3′; 5′ tagcAAGCTTctatgaccacgcagatggcttgtcagttaaaagaaaACTAGTagtc 3′. The reporter assays were performed as previously described (Chen et al., 2015). In brief, NCCs were co-transfected with 200 ng of constructed plasmids containing miR-135a binding sites, 20 ng Renilla luciferase pRL-TK control reporter vector (Promega, Madison, WI, United States) and 50 nM of miRNA-135a mimics or mimic control (Ambion, Austin, TX, United States) using Lipofectamine 2000 (Invitrogen, Carlsbad, CA, United States) according to the manufacturer’s protocol. Luciferase activity was then measured at 48 h after the transfection using the Dual-Luciferase assay kit (Promega, Madison, WI, United States) with a Lumat LB 9507 Ultra Sensitive Tube Luminometer (Berthold Technologies, Bad Wildbad, Germany). The luciferase activity of each sample was normalized to the pRL/TK-driven Renilla luciferase activity.

### Western Blotting

Western blotting was performed by standard protocols as described previously (Chen et al., 2013b; Sun et al., 2014). Proteins were probed with the following antibodies: SIAH1 rabbit pAb (Abcam, Cambridge, MA, United States), β-Actin mouse mAb (Santa Cruz, Santa Cruz, CA, United States), Phospho-p38 MAPK (Thr180/Tyr182) rabbit mAb (Cell Signaling Technology, Inc., Beverly, MA, United States), p38 MAPK rabbit pAb (Cell Signaling Technology, Inc., Beverly, MA, United States), Phospho-p53 (Ser15) rabbit pAb (Cell Signaling Technology, Inc., Beverly, MA, United States), p53 mouse mAb (Abcam, Cambridge, MA, United States), p53 upregulated modulator of apoptosis (PUMA) rabbit pAb (Abcam, Cambridge, MA, United States), Bcl-2 homologous antagonist/killer (Bak) rabbit pAb (Cell Signaling Technology, Inc., Beverly, MA, United States), and cleaved caspase-3 (Asp175) rabbit pAb (Cell Signaling Technology, Inc., Beverly, MA, United States). The membranes were developed on Molecular Imager ChemiDoc XRS + (Bio-Rad, Hercules, CA, United States), and the intensity of the protein band was analyzed by ImageJ software (1.48V, National Institutes of Health, United States). All Western blot analyses were performed in triplicate.

### Analysis of Apoptosis

Apoptosis was determined by the analysis of caspase-3 cleavage and activity, as well as the terminal deoxynucleotidyl transferase dUTP nick end labeling (TUNEL) assay. Caspase-3 cleavage was determined by Western blot as described previously (Dong et al., 2008; Chen et al., 2013a). Caspase-3 activity was determined by using Caspase-Glo^®^ 3/7 Assay Systems (Promega, Madison, WI, United States). TUNEL assay was performed by using a TiterTACS *In situ* Detection Kit (Trevigen, Inc., Gaithersburg, MD, United States), following the manufacturer’s protocol.

### Morphological Analysis and Whole-Mount Skeletal Staining

Morphological analysis of zebrafish larvae at 4 dpf was performed by using a stereoscopic microscope (Olympus SZX16, Tokyo, Japan). Whole-mount skeletal staining of zebrafish larvae at 5 dpf was conducted with Alcian blue staining (Sigma Chemical, Co., St Louis, MO, United States), as described by others (Neuhauss et al., 1996; Walker and Kimmel, 2007), and was visualized by a stereoscopic microscope (Olympus SZX16, Tokyo, Japan) as previously described (Carvan et al., 2004).

### Statistical Analysis

Statistical analyses were performed as described previously (Chen et al., 2013b) using GraphPad Prism software (GraphPad Software, San Diego, CA, United States). All data were expressed as means ± SD of three separate experiments. One-way ANOVA was used to compare the difference between groups, and multiple comparison post-tests were conducted by using Bonferroni’s test. Differences between groups were considered significant at *p* < 0.05.

## Results

### Ethanol Treatment Significantly Decreased the Expression of miR-135a in NCCs and Zebrafish Embryos

**FIGURE 1:**
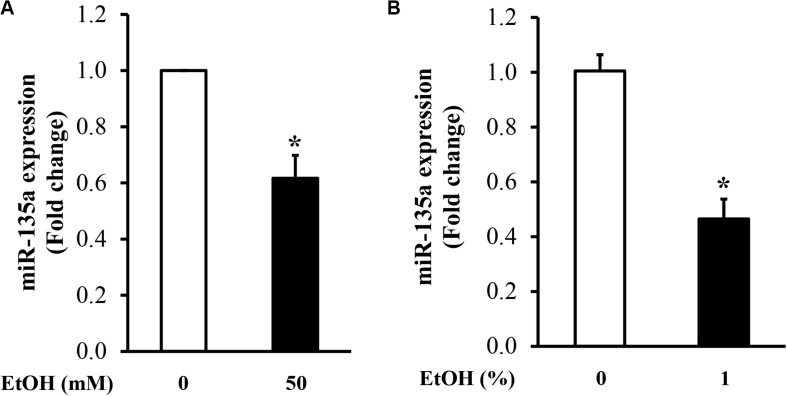
Ethanol exposure decreased the expression of miR-135a in neural crest cells (NCCs) exposed to 50 mM ethanol for 24 h **(A)** and zebrafish embryos treated with 1% ethanol and collected at 24 h post-fertilization (hpf; **B**). The expression of miR-135a was determined by qRT-PCR, as described in the section “Materials and Methods”. Data are expressed as fold change over control and represent the mean ± SD of three separate experiments. * *p* < 0.05 vs. control.

To determine the effect of ethanol treatment on the expression of miRNA-135a in NCCs, NCCs were exposed to 50 mM ethanol for 24 h. MiR-135a expression level was examined by qRT-PCR. As shown in Figure 1A, exposure of NCCs to 50 mM ethanol for 24 h significantly decreased the expression of miR-135a in NCCs, indicating that ethanol treatment can downregulate the expression of miR-135a in NCCs. To determine whether ethanol exposure can also decrease the expression of miR-135a *in vivo*, zebrafish embryos at 3 hpf were treated with or without 1% ethanol and collected at 24 hpf for analysis of miR-135a expression using the qRT-PCR assay. As shown in Figure 1B, 1% ethanol treatment resulted in a significant reduction of miR-135a expression in zebrafish embryos. These results demonstrate that both *in vitro* and *in vivo* ethanol treatment can decrease the expression of miR-135a.

### Siah1 Is the Direct Target of miR-135a in NCCs

**FIGURE 2:**
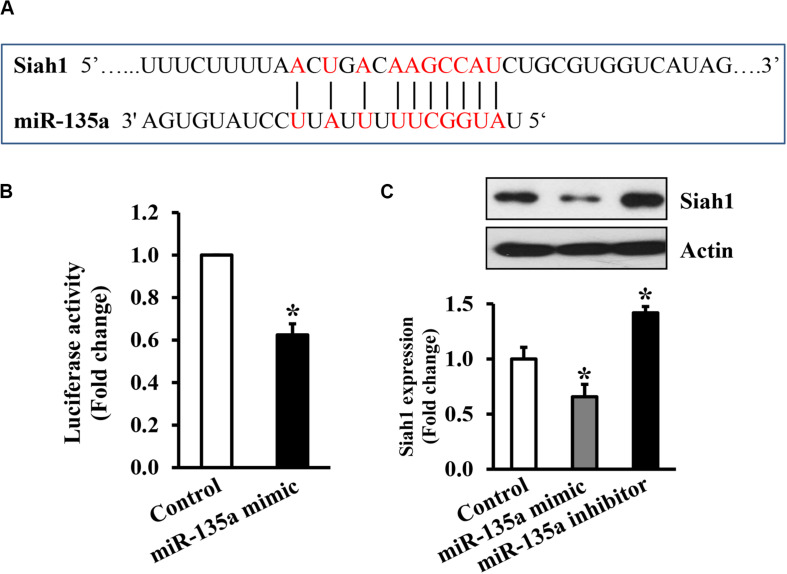
Siah1 is the direct target of miR-135a in neural crest cells (NCCs). **(A)** The predicted binding sites of miR-135a in the 3′-UTR of Siah1 mRNA. **(B)** Luciferase reporter assays for the binding of miR-135a to the 3′-UTR of Siah1 in NCCs. **(C)** Western bolt analysis of the protein expression of Siah1 in NCCs transfected with miR-135a mimics or inhibitors for 48 h. Data are expressed as fold change over control and represent the mean ± SD of three separate experiments. * *p* < 0.05 vs. control.

Bioinformatics prediction indicated that Siah1 is a direct target of miR-135a (Figure 2A). Siah1 has also been validated to be a direct target of miRNA-135a in HeLa cells (Pang et al., 2011). To validate that Siah1 is also a direct target of miR-135a in NCCs, we cloned the 3′-UTR segments of Siah1 into the pMIR-Report vector to create a luciferase reporter system. NCCs were co-transfected with the vector containing pMIR reporter-luciferase fused with or without the 3′-UTR of Siah1 and miR135a mimic or miRNA control mimic. As shown in Figure 2B, co-transfected of the 3′-UTR of Siah1 mRNA and miR135a resulted in a significant reduction in luciferase activity as compared to the NCCs co-transfected with Siah1 3′-UTR and control miRNA mimic. In addition, overexpression of miR-135a by transfecting with miR-135a mimics greatly downregulated the protein expression of Siah1, while downregulation of endogenous miR-135a through transfecting with miR-135a inhibitors significantly elevated the protein expression of Siah1 (Figure 2C), demonstrating that Siah1 is a direct target of miR-135a in NCCs.

### Over-Expression of miR-135a Diminished Ethanol-Induced Upregulation of Siah1 in NCCs and Zebrafish Embryos

**FIGURE 3:**
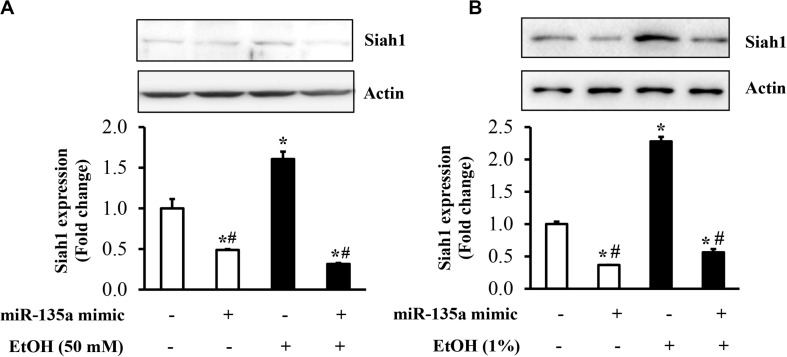
Overexpression of miR-135a significantly decreased the ethanol-induced upregulation of Siah1 in neural crest cells (NCCs) and zebrafish embryos. NCCs transfected with control mimics or miR-135a mimics were treated with 50 mM ethanol for 24 h. Zebrafish embryos microinjected with control or miR-135a mimics were treated with 1% ethanol and collected at 24 h post-fertilization (hpf). The protein expression of Siah1 was examined in NCCs **(A)** and zebrafish embryos **(B)** by western blot. Data are expressed as fold change over control and represent the mean ± SD of three separate experiments. * *p* < 0.05 vs. control; # *p* < 0.05 vs. ethanol.

To determine whether the downregulation of miR-135a contributes to ethanol-induced upregulation of Siah1, NCCs transfected with miR-135a mimics or control mimics were exposed to 50 mM ethanol, and the protein expression of Siah1 was analyzed. As shown in Figure 3A, ethanol exposure resulted in a significant increase in the protein expression of Siah1. Overexpression of miR-135a by transfection with miR-135a mimics diminished ethanol-induced upregulation of Siah1 in NCCs, demonstrating that downregulation of miR-135a contributes to ethanol-induced upregulation of Siah1 in NCCs. To determine whether overexpression of miR-135a can also diminish ethanol-induced upregulation of Siah1 in zebrafish embryos, zebrafish embryos microinjected with miR-135a mimics and control mimics were exposed to 1% ethanol, and the protein expression of Siah1 was analyzed by western blot. As shown in Figure 3B, exposure to ethanol significantly increased the expression of Siah1. Microinjection of miR-135a mimics significantly diminished ethanol-induced upregulation of Siah1 in zebrafish embryos, indicating that overexpression of miR-135a can also prevent ethanol-induced upregulation of Siah1 in zebrafish embryos.

### Overexpression of miR-135a Diminished Ethanol-Induced Activation of the p38 MAPK/p53 Pathway in NCCs

**FIGURE 4:**
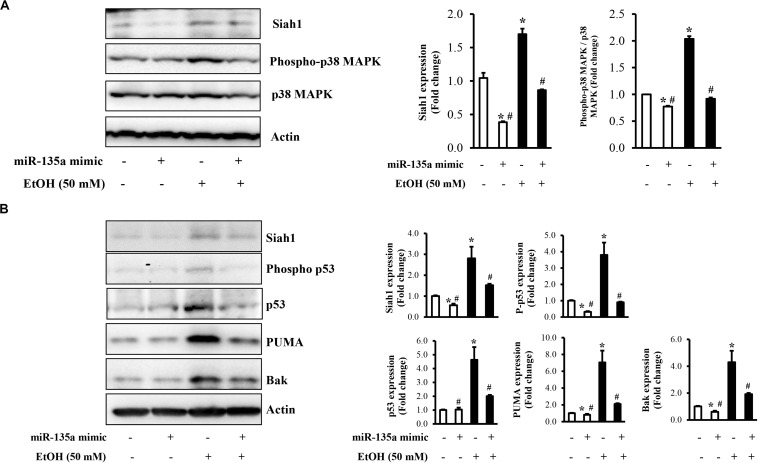
Overexpression of miR-135a reduced the ethanol-induced activation of the p38 mitogen-activated protein kinase (MAPK)/p53 pathway in neural crest cells (NCCs). NCCs transfected with control mimics or miR-135a mimics were treated with 50 mM ethanol for 24 h. The expression of Siah1, total p38, and phosphorylated p38 **(A)**, and the expression of Siah1, phosphorylated p53, p53, p53 upregulated modulator of apoptosis (PUMA), and Bcl-2 homologous antagonist/killer (Bak; **B**) were determined by western blot. Data are expressed as fold change over control and represent the mean ± SD of three separate experiments. * *p* < 0.05 vs. control; # *p* < 0.05 vs. ethanol.

Our previous study has demonstrated that ethanol-induced upregulation of Siah1 triggered apoptosis in NCCs through promoting p38 MAPK-mediated activation of the p53 signaling pathway (Yuan et al., 2017). To determine whether the downregulation of miR-135a contributes to ethanol-induced apoptosis in NCCs through Siah1-mediated activation of the p38 MAPK/p53 pathway, we first examined whether the overexpression of miRNA-135a can inhibit the activation of the p38 MAPK pathway in ethanol-exposed NCCs. As shown in Figure 4A, ethanol treatment resulted in the activation of the p38 MAPK pathway, as indicated by enhanced phosphorylation of p38 MAPK, consisting with our previous studies (Yuan et al., 2017). Overexpression of miR-135a significantly diminished ethanol-induced expression of phosphor-p38 MAPK, indicating that miR-135a can inhibit the p38 MAPK pathway through downregulating Siah1. To determine the role of miRNA-135a on ethanol-induced apoptosis in NCCs, we next examined the p53 apoptotic pathway, which is the downstream targets of the p38 MAPK pathway that has been shown to be regulated tightly by Siah1 in NCCs (Yuan et al., 2017). We found that ethanol exposure increased the total protein levels of p53 and the levels of the phosphorylated p53. Ethanol treatment also significantly increased the expression of p53 downstream targets, PUMA and Bak. Overexpression of miRNA-135a significantly diminished ethanol-induced increases in the phosphorylation and total protein level of p53, and the expression of PUMA and Bak (Figure 4B), indicating that miRNA-135a can modulate the p38 MAPK/p53 apoptotic pathway in NCCs through targeting Siah1.

### Overexpression of miR-135a Significantly Reduced Ethanol-Induced Activation of Caspase-3 and Diminished Ethanol-Induced Apoptosis in NCCs and Zebrafish Embryos

**FIGURE 5:**
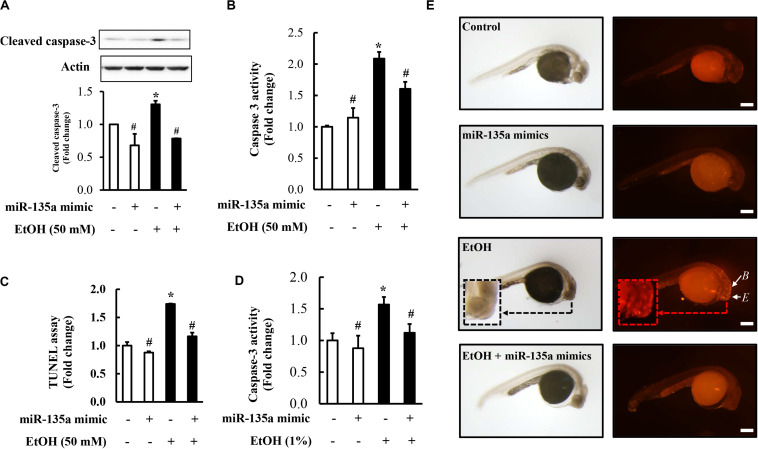
Overexpression of miR-135a significantly decreased ethanol-induced caspase-3 activation and apoptosis in neural crest cells (NCCs) and zebrafish embryos. NCCs transfected with control mimics or miR-135a mimics were treated with 50 mM ethanol for 24 h. Zebrafish embryos microinjected with control or miR-135a mimics were treated with 1% ethanol and collected at 24 h post-fertilization (hpf). Ethanol-induced apoptosis in NCCs **(A-C)** and zebrafish embryos **(D,E)** was determined by analysis of caspase-3 cleavage **(A)**, caspase-3 activity **(B,D)**, and terminal deoxynucleotidyl transferase dUTP nick end labeling (TUNEL) analysis **(C,E).** White arrows in **(E)** indicate brain **(B)** and eye **(E)** in zebrafish embryos. Data are expressed as fold change over control and represent the mean ± SD of three separate experiments. * *p* < 0.05 vs. control; # *p* < 0.05 vs. ethanol. Scale bar = 200 μm.

To determine whether overexpression of miR-135a can prevent ethanol-induced apoptosis in NCCs, NCCs transfected with miR-135a mimics were exposed to 50 mM ethanol for 24 h. As shown in Figures 5A,B, ethanol exposure resulted in a significant increase in caspase-3 cleavage and activity in NCCs, indicating that ethanol exposure can increase apoptosis in NCCs that was confirmed by the TUNEL assay (Figure 5C). Overexpression of miR-135a significantly diminished ethanol-induced increases in caspase-3 cleavage and activity and reduced apoptosis in ethanol-exposed NCCs (Figures 5A–C). These results demonstrate that overexpression of miR-135a can prevent ethanol-induced apoptosis in NCCs. To determine whether the upregulation of miR-135a can also prevent ethanol-induced apoptosis in zebrafish embryos, zebrafish embryos microinjected with miR-135a mimics and control mimics were exposed to ethanol from 3 to 24 hpf. As shown in Figure 5D, microinjection of miR-135a mimics significantly reduced ethanol-induced increase in caspase-3 activity in zebrafish embryos. Whole-mount TUNEL staining also shown that ethanol-induced apoptosis was significantly attenuated by the microinjection of miR-135a mimics in zebrafish embryos, especially in the brain and eye (Figure 5E).

### Microinjection of miR-135a Mimics Attenuated Ethanol-Induced Growth Retardation and Dysmorphology in Zebrafish Larvae

**FIGURE 6:**
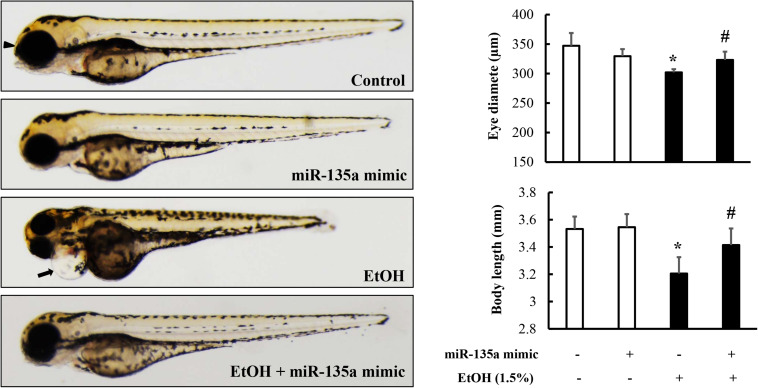
Microinjection of miR-135a mimics diminished ethanol-induced growth retardation and dysmorphology in zebrafish larvae. Embryos microinjected with control or miR-135a mimics were treated with 1.5% ethanol from 3 to 24 h post-fertilization (hpf). Zebrafish larvae were collected at 4 days post-fertilization (dpf) for morphological analysis. Analysis of ethanol-induced growth retardation and dysmorphology was performed by using a stereoscopic microscope. The black arrows indicate the heart and the arrowheads indicate eye. Data represent the mean ± SD of three separate experiments. * *p* < 0.05 vs. control; # *p* < 0.05 vs. ethanol.

It has been reported that ethanol-induced apoptosis in NCCs contributes heavily to the subsequent abnormalities that are characteristics of FASD (Kotch and Sulik, 1992a; Cartwright and Smith, 1995a). To determine whether microinjection of miR-135a mimics can attenuate ethanol-induced growth retardation and dysmorphology in zebrafish, zebrafish embryos microinjected with control or miR-135a mimics were treated with 1.5% ethanol from 3 to 24 hpf and then were allowed to grow in system water without ethanol. Zebrafish larvae from control and treated groups were collected at 4 dpf for morphological analysis. As shown in Figure 6, ethanol treatment resulted in significant growth retardation in zebrafish larvae, as indicated by the dramatically reduced body length. Ethanol exposure also resulted in significant developmental defects, including small eyes, microcephaly, and pericardial edema. Microinjection of miR-135a mimics significantly attenuated the ethanol-induced growth retardation and dysmorphology in zebrafish larvae.

### Microinjection of miR-135a Mimics Significantly Diminished Ethanol-Induced Craniofacial Cartilage Defects in Zebrafish Larvae

**FIGURE 7:**
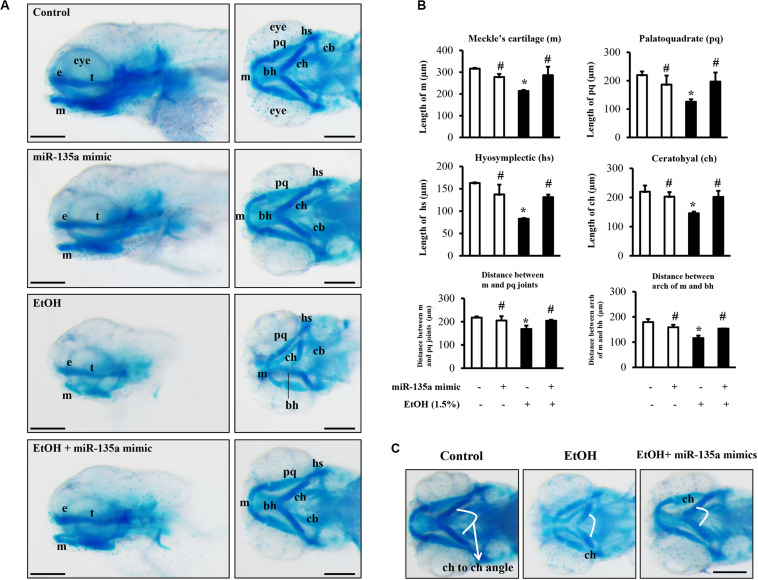
Microinjection of miR-135a mimics attenuated ethanol-induced craniofacial cartilage defects in zebrafish larvae. Embryos microinjected with control or miR-135a mimics were treated with 1.5% ethanol from 3 h post-fertilization (hpf) to 24 hpf. Zebrafish larvae were collected at 5 days post-fertilization (dpf) for skeletal staining with Alcian blue. **(A)** Lateral and ventral view of Alcian blue-stained craniofacial cartilages in larvae from different treatment groups. **(B)** Morphometric analysis of the craniofacial cartilages in larvae from different treatment groups. The length of Meckel’s cartilage (m), palatoquadrate (pq), hyosymplectic (hs), ceratohyal (ch), and the distance between m and pq joint, and between the arch of m and basihyal (bh) were measured. **(C)** Comparisons of ceratohyal cartilage (ch) angle between different treatment groups. Data represent the mean ± SD of three separate experiments. * *p* < 0.05 vs. control; # *p* < 0.05 vs. ethanol. e, ethmoid plate; t, trabeculae cranii; cb, ceratobranchial; bh, basihyal. Scale bar = 200 μm.

To determine whether the microinjection of miR-135a mimics can attenuate the ethanol-induced craniofacial cartilage defects in zebrafish larvae, zebrafish embryos microinjected with control or miRNA-135a mimics were treated with 1.5% ethanol from 3 to 24 hpf. Zebrafish larvae were collected at 5 dpf and stained with Alcian blue. As shown in Figure 7A, ethanol exposure resulted in an overall reduction in head size and micrognathia due to reduced jaw outgrowth in zebrafish larvae. A closer examination of craniofacial cartilage at 5 dpf larvae revealed that the length of the mandibular arch cartilages (lower jaw), including the ventral Meckel’s cartilage (m) and dorsal palatoquadrate (pq), were significantly reduced in ethanol-exposed zebrafish larvae, as compared to control. In addition, exposure to ethanol at the embryonic stage significantly reduced the length of hyosymplectic (hs) and ceratohyal (ch), and the distance between m and pq joint and between the arch of m and basihyal (bh; Figure 7B). Embryonic ethanol treatment also led to the abnormal angulation of the ch cartilage (Figure 7C). Overexpression of miR-135a significantly diminished ethanol-induced reduction in the length of m, pq, hs, and ch and the distance between m and pq joints and between m and bh. Microinjection of miR-135a also attenuated the ethanol-induced abnormal angulation of ch cartilage, indicating that the upregulation of miR-135a can attenuate ethanol-induced craniofacial cartilage defects in zebrafish larvae (Figures 7A–C).

## Discussion

Growing evidence suggests that impairment of differentiation, migration, and survival of NCCs is a major component of the pathogenesis of FASD and that dysregulation of gene expression is a key driver of the ethanol-induced impairment in NCCs (Kotch and Sulik, 1992b; Cartwright and Smith, 1995b; Rovasio and Battiato, 2002; Steventon et al., 2014; Zhang et al., 2017; Yuan et al., 2018; Fan et al., 2019; Li et al., 2019). MicroRNAs have been shown to be involved in the ethanol-induced impairment of NCCs. Our previous studies have shown that miR-125b can protect against ethanol-induced apoptosis in NCCs and mouse embryos by targeting Bak1 and PUMA and that microinjection of miR-125b mimics can prevent ethanol-induced embryotoxicity (Chen et al., 2015). We have also demonstrated that miR-34a can mediate ethanol-induced impairment of neural differentiation of NCCs by targeting autophagy-related gene 9a (Fan et al., 2019). In the present study, we found that ethanol exposure significantly reduced the expression of miR-135a, increased apoptosis in NCCs exposed to 50 mM ethanol and zebrafish embryos exposed to 1.0% ethanol, and resulted in growth retardation and developmental defects in zebrafish larvae exposed to 1.5% ethanol. There is a consensus that the ethanol levels in the zebrafish embryo tissue are approximately 25–35% of the levels of the medium (Flentke et al., 2014; Lovely et al., 2014). Therefore, 1.0 or 1.5% ethanol in the medium is ∼ 300 or 450 mg/dL in the embryo tissue. These ethanol concentrations were chosen because studies have shown that a peak maternal blood alcohol concentration of 400–500 mg/dL (approximately 85–105 mM) is needed to produce major malformations with the characteristics of fetal alcohol syndrome in mouse embryos (Sulik et al., 1981; Kotch and Sulik, 1992a; Dunty et al., 2001). These ethanol concentrations are relatively high but are not beyond that which can be observed in chronic alcoholics (Adachi et al., 1991).

MiR-135a is a member of the miR-135 superfamily. A number of studies have reported that the downregulation of miR-135a was involved in tumorigenesis in a variety of cancers, in which miR-135a exerts tumor-suppressive effects (Yamada et al., 2013; Kroiss et al., 2015; Zhao et al., 2017). In contrast, other studies have shown that miR-135a is an oncogenic miRNA in colorectal carcinomas (Nagel et al., 2008) and that overexpression of miR-135a promoted tumorigenesis of portal vein tumor thrombus (Liu et al., 2012) and enhanced the growth of HeLa- and NC104-E6/E7-derived tumor (Leung et al., 2014). Studies have also shown that miR-135a is involved in brain inflammation and angiogenesis in Alzheimer’s disease (Ko et al., 2015). In addition, it has been reported that miR-135a can promote proliferation and prevent apoptosis. For example, a study by Dong et al., indicated that, by targeting HIF-1α, miR-135a downregulated pro-apoptotic genes Bax and Bad, upregulated anti-apoptotic gene Bcl-2, resulting in increased proliferation and reduced apoptosis of astrocytes in the bacterial meningitis rat models (Dong et al., 2019). Upregulation of miR-135a also protected human umbilical vein endothelial cells against mechanical stretch-induced increases in apoptosis and ventilator-induced lung injury through activating PI3K/Akt signaling pathway by targeting PH domain leucine-rich repeat-containing protein phosphatase 2 (PHLPP2) (Yan et al., 2018). In the present study, we have shown that ethanol-induced downregulation of miR-135a resulted in the upregulation of Siah1 and that overexpression of miR-135a significantly reduced ethanol-induced upregulation of Siah1, indicating that downregulation of miR-135a contributes to ethanol-induced upregulation of Siah1 in NCCs and zebrafish embryos.

Siah is a member of a highly conserved family of E3 ubiquitin ligases (Carthew and Rubin, 1990). Siah ligases are involved in the ubiquitination and proteasomal degradation of several proteins that are essential for a variety of signaling pathways, including membrane receptors (Liani et al., 2004; Winter et al., 2008), a microtubule-associated motor protein (Linares-Cruz et al., 1998) and transcriptional regulators (Zhang et al., 1998; Tiedt et al., 2001). The upregulation of Siah1 has also been found to induce cell-cycle arrest and the induction of apoptosis (Relaix et al., 2000; Matsuzawa and Reed, 2001). We have also demonstrated that ethanol treatment can significantly increase the expression and nuclear translocation of Siah1 in NCCs and that Siah1 signaling plays a critical role in ethanol-induced apoptosis in NCCs (Sun et al., 2014). In addition, we have shown that ethanol-induced up-regulation of Siah1 can induce apoptosis in NCCs through p38 MAPK-mediated activation of the p53 signaling pathway (Yuan et al., 2017). In this study, we found that, in addition to NCCs, ethanol exposure can also increase the expression of Siah1 in zebrafish embryos. We also found that ethanol-induced upregulation of Siah1 resulted in the activation of p38 MAPK/p53 pathway and that overexpression of miR-135a significantly diminished ethanol-induced activation of p38 MAPK/p53 pathway. These results consist of the findings from our previous study and demonstrate that the upregulation of miR-135a can inhibit p38 MAPK-mediated activation of the p53 signaling pathway through downregulating Siah1.

It is well known that p38 MAPK pathway can modulate apoptosis by regulating p53 pathway (Zheng et al., 2005; Gao et al., 2014) and that p53 is a transcription factor that regulates the expression of genes involved in apoptosis (Kho et al., 2004; Schuler and Green, 2005). Previous studies from our laboratory have shown that knockdown of Siah1 by siRNA significantly diminished the ethanol-induced increase in the phosphorylation of p38 MAPK, and significantly decreased ethanol-induced increases in p53 stability and its phosphorylation in NCCs, and that p38 MAPK activation is essential for ethanol-induced Siah1-mediated p53 activation, leading to apoptosis in ethanol-exposed NCCs (Yuan et al., 2017). In this study, we found that ethanol treatment significantly increased the expression of p53 downstream targets, PUMA and Bak, the activation of caspase-3 and apoptosis in NCCs. Overexpression of miRNA-135a significantly diminished ethanol-induced upregulation of PUMA and Bak and apoptosis in NCCs. In addition, microinjection of miR-135a mimics dramatically reduced the apoptosis in zebrafish embryos and diminished ethanol-induced growth retardation and dysmorphology in zebrafish larvae. These results demonstrate that the upregulation of miR-135a can prevent ethanol-induced apoptosis in NCCs and developmental defects in zebrafish embryos by modulating the Siah1-mediated p38 MAPK/p53 pathway.

## Conclusion

The present study has demonstrated that ethanol treatment decreased the expression of miR-135a and thereby increased the expression of its direct target, Siah1, which, in turn, activated the p38 MAPK/p53 pathway, increased apoptosis in NCCs and zebrafish embryos and resulted in growth retardation and developmental defects. Overexpression of miR-135a significantly reduced ethanol-induced upregulation of Siah1 and the activation of the p38 MAPK/p53 pathway, decreased ethanol-induced apoptosis in NCCs and zebrafish embryos, and diminished ethanol-induced growth retardation and dysmorphology in zebrafish larvae. These results demonstrate that ethanol-induced downregulation of miR-135a contributes to ethanol-induced apoptosis in NCCs and craniofacical defects in zebrafish embryos by upregulating Siah1 and activating the p38 MAPK/p53 pathway. These findings elucidate the mechanisms by which miR-135a modulates ethanol-induced apoptosis in NCCs and craniofacial defects in zebrafish embryos and suggest that miR-135a may represent a novel therapeutic target for the intervention and prevention of FASD.

## Data Availability Statement

All datasets generated in this study are included in the article.

## Ethics Statement

The animal study was reviewed and approved by Institutional Animal Care and Use Committee of the University of Louisville.

## Author Contributions

FY and SC conceptualized and designed the experiments and participated in data interpretation and manuscript preparation. FY, YY, and JL performed the experiments and participated in data analysis. HF, YL, LL, and WF participated in data interpretation and discussion. All authors reviewed the manuscript.

## Conflict of Interest

The authors declare that the research was conducted in the absence of any commercial or financial relationships that could be construed as a potential conflict of interest.

## Footnotes

1 http://www.targetscan.org/

2 http://www.microrna.org/microrna/home/do

## Abbreviations

miRNA: microRNA
FASD: fetal alcohol spectrum disorders
NCCs: neural crest cells
Siah1: seven *in absentia* homolog 1
Bcl-2: B-cell lymphoma 2
Nrf2: nuclear factor (erythroid-derived 2)-like 2
MAPK: mitogen-activated protein kinase
3′-UTRs: 3′-untranslated region
hpf: hours post-fertilization
dpf: days post fertilization
TUNEL: terminal deoxynucleotidyl transferase dUTP nick end labeling
Bak: Bcl-2 homologous antagonist/killer
PUMA: p53 upregulated modulator of apoptosis
m: Meckel’s cartilage
e: ethmoid plate
t: trabeculae cranii
pq: palatoquadrate
hs: hyosymplectic
ch: ceratohyal
cb: ceratobranchial
bh: basihyal.
